# Supplementary material for: Genetic Manipulation of the Brassicaceae Smut Fungus Thecaphora thlaspeos
Source: J Fungi (Basel). 2021 Jan 9;7(1):38. doi: 10.3390/jof7010038 (PMC7826943; doi:10.3390/jof7010038)
Supplement: Supplementary file 1 [file jof-07-00038-s001.zip › Supplementary Files_proofs/FigS1_buffer_comparison.pdf]

Figure S1

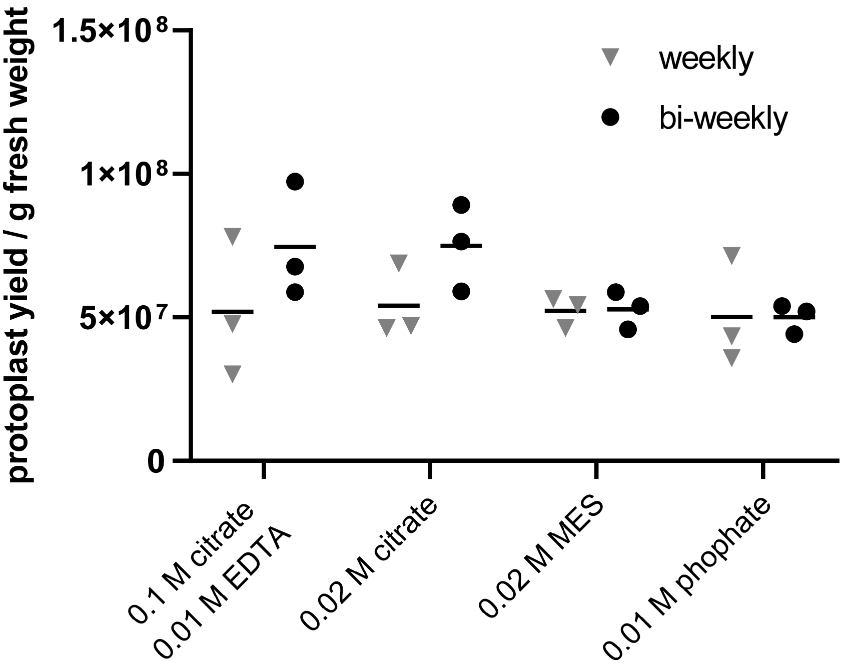

Fig. S1: Protoplast yields in different buffers are influenced by the sub-culturing scheme. *T. thlaspeos* LF1 cultures were grown to an OD600<0.8 in an either weekly or bi-weekly splitting rhythm. To test protoplasting efficiency, hyphae were filtered and incubated in different buffers all supplemented with 1.2 M MgSO4 and 10 mg/ml Yatalase + 20 mg/ml Glucanex for 60 mins at RT. There are no significant differences between buffers or splitting rhythm of the culture, but cells sub-cultured bi-weekly yield more protoplasts in citrate buffers.
