# Supplementary material for: Genetic Manipulation of the Brassicaceae Smut Fungus Thecaphora thlaspeos
Source: J Fungi (Basel). 2021 Jan 9;7(1):38. doi: 10.3390/jof7010038 (PMC7826943; doi:10.3390/jof7010038)
Supplement: Supplementary file 1 [file jof-07-00038-s001.zip › Supplementary Files_proofs/FigS2_antibiotics.pdf]

## Figure S2

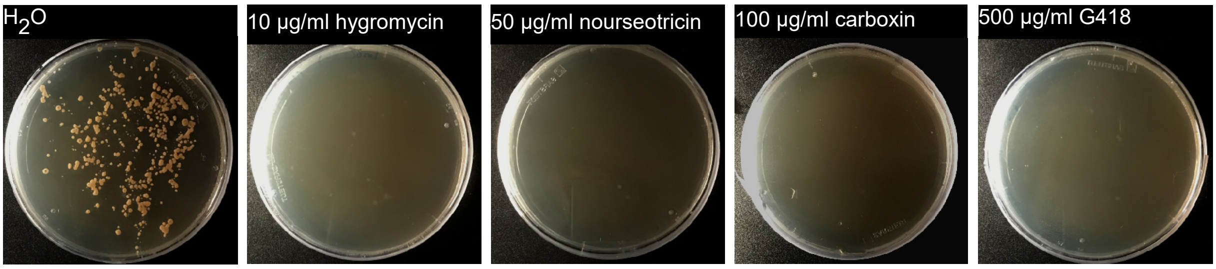


**Figure S2: Sensitivity of *T. thlaspeos* towards different antibiotics.** LF1 was grown on YL 0.6% plant agar plates with and without antibiotics for 16 days. For hygromycin and nourseotricin dilution series were tested and 10 µg hygromycin and 50 µg nourseotricin were identified as minimal inhibitory concentrations to prevent growth. Carboxin inhibits growth at 100 µg/ml and G418 at 500 µg/ml. Both can be used to establish additional resistance mark-ers.
