# Supplementary material for: Genetic Manipulation of the Brassicaceae Smut Fungus Thecaphora thlaspeos
Source: J Fungi (Basel). 2021 Jan 9;7(1):38. doi: 10.3390/jof7010038 (PMC7826943; doi:10.3390/jof7010038)
Supplement: Supplementary file 1 [file jof-07-00038-s001.zip › Supplementary Files_proofs/FigS3_cbx_alignment.pdf]

Figure S3

```
UmSDH2 sen  MSLFNVSNGL RTALRPSVAS S----SRVAA FSTTAAARLA TPTSD-NVGS SGKPQHLKQF KIYRW 60
UmSDH2 res  .......... .......... .----..... .......... .....-.... .......... ..... 60
THTG_04978  ....-AG.T. .A.VQ.V.SG .RSSIA.T.. .....L.Q.. .....S.... .....K..A. ..... 64

UmSDH2 sen  NPDKPSEKPR LQSYTLDLNQ TGPMVLDALI KIKNEIDPTL TFRRSCREGI CGSCAMNIDG VNTLA 125
UmSDH2 res  .......... .......... .......... .......... .......... .......... ..... 125
THTG_04978  .....A.... ..T....... .......... .....V..S. .......... .......... ..... 129

UmSDH2 sen  CLCRIDKQND TKIYPLPHMY IVKDLVPDLT QFYKQYRSIE PFLKSNNTPS EGEHLQSPEE RRRLD 190
UmSDH2 res  .......... .......... .......... .......... .......... .......... ..... 190
THTG_04978  ......REKE S......... V......... .......... .....K.P.A Q......... ..... 194

UmSDH2 sen  GLYECILCAC CSTSCPSYWW NQDEYLGPAV LMQAYRWMAD SRDDFGEERR QKLENTFSLY RCHTI 255
UmSDH2 res  .......... .......... .......... .......... .......... .......... ..L.. 255
THTG_04978  .......... .......... .......... .......... .....T...K T......... A.R.. 259

UmSDH2 sen  MNCSRTCPKN LNPGKAIAQI KKDMAVGAP- KASERPIMAS S 295
UmSDH2 res  .......... .......... .........- .......... . 295
THTG_04978  ...T.....S ...A....T. ..E.ST...A .STD....P. N 300
```

Fig. S3: Sequence comparison of the succinate dehydrogenase. *Um*Sdh2 (UMAG_00844) exists in two isoforms that differ in only one amino acid (marked in grey). The carboxin-resistant version carries a leucine instead of a histidine in position 253 (Keon et al., 1991 [41]). *T. thlaspeos* has a homolog that has 82 % amino acid similarity, but has an arginine in the resistance-mediating position. This difference might explain the lower sensitivity against carboxin compared to *U. maydis*.
