# Supplementary material for: Genetic Manipulation of the Brassicaceae Smut Fungus Thecaphora thlaspeos
Source: J Fungi (Basel). 2021 Jan 9;7(1):38. doi: 10.3390/jof7010038 (PMC7826943; doi:10.3390/jof7010038)
Supplement: Supplementary file 1 [file jof-07-00038-s001.zip › Supplementary Files_proofs/FigS4_transformation_constructs_in_U_maydis.pdf]

Figure S4

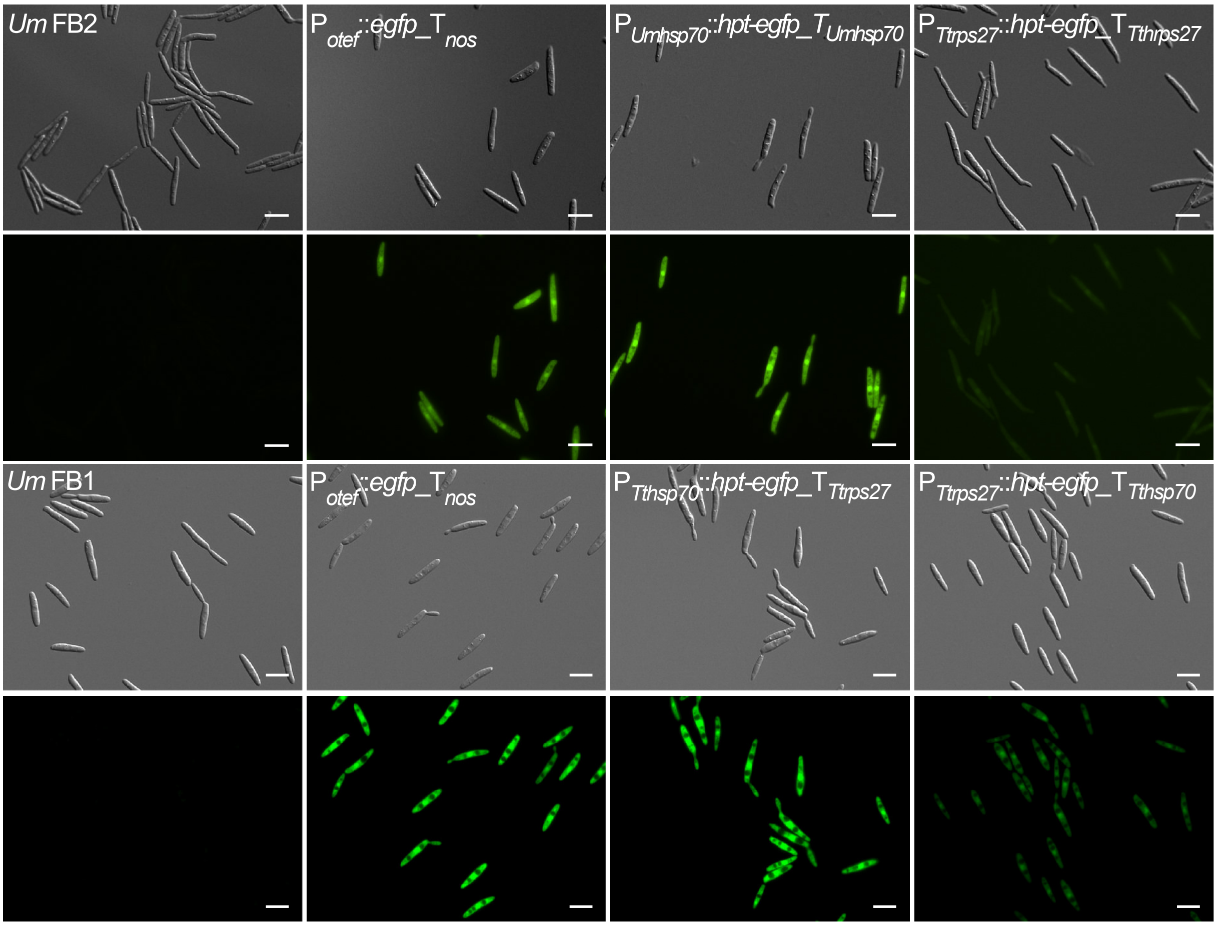

Figure S4: Verification of resistance-reporter constructs in *U. maydis*. Reporter constructs containing a fusion of hygromycin resistance (*hpt*) and the fluorescent marker *egfp* under the control of two candidate promoter regions derived from the *T. thlaspeos* genome were tested in *U. maydis* wild-type strains. Upon transformation of the linearized construct, it randomly integrates into the genome. Protein accumulation is visualized by green fluorescence. eGfp-fluorescence in the strains carrying the promoter of *Tthsp70* was stronger compared to the stably integrated construct under the control of a strong, synthetic promoter (P*otef*). Expression under the promoter region of the ribosomal gene *rsp27* was weaker, but the cells still were resistant to hygromycin. This confirms that the fusion protein is active and both promoters can be used, albeit with different strength. Scale bar: 10 µm.
