# Supplementary material for: Genetic Manipulation of the Brassicaceae Smut Fungus Thecaphora thlaspeos
Source: J Fungi (Basel). 2021 Jan 9;7(1):38. doi: 10.3390/jof7010038 (PMC7826943; doi:10.3390/jof7010038)
Supplement: Supplementary file 1 [file jof-07-00038-s001.zip › Supplementary Files_proofs/FigS5_strain_confirmation.pdf]

Figure S5

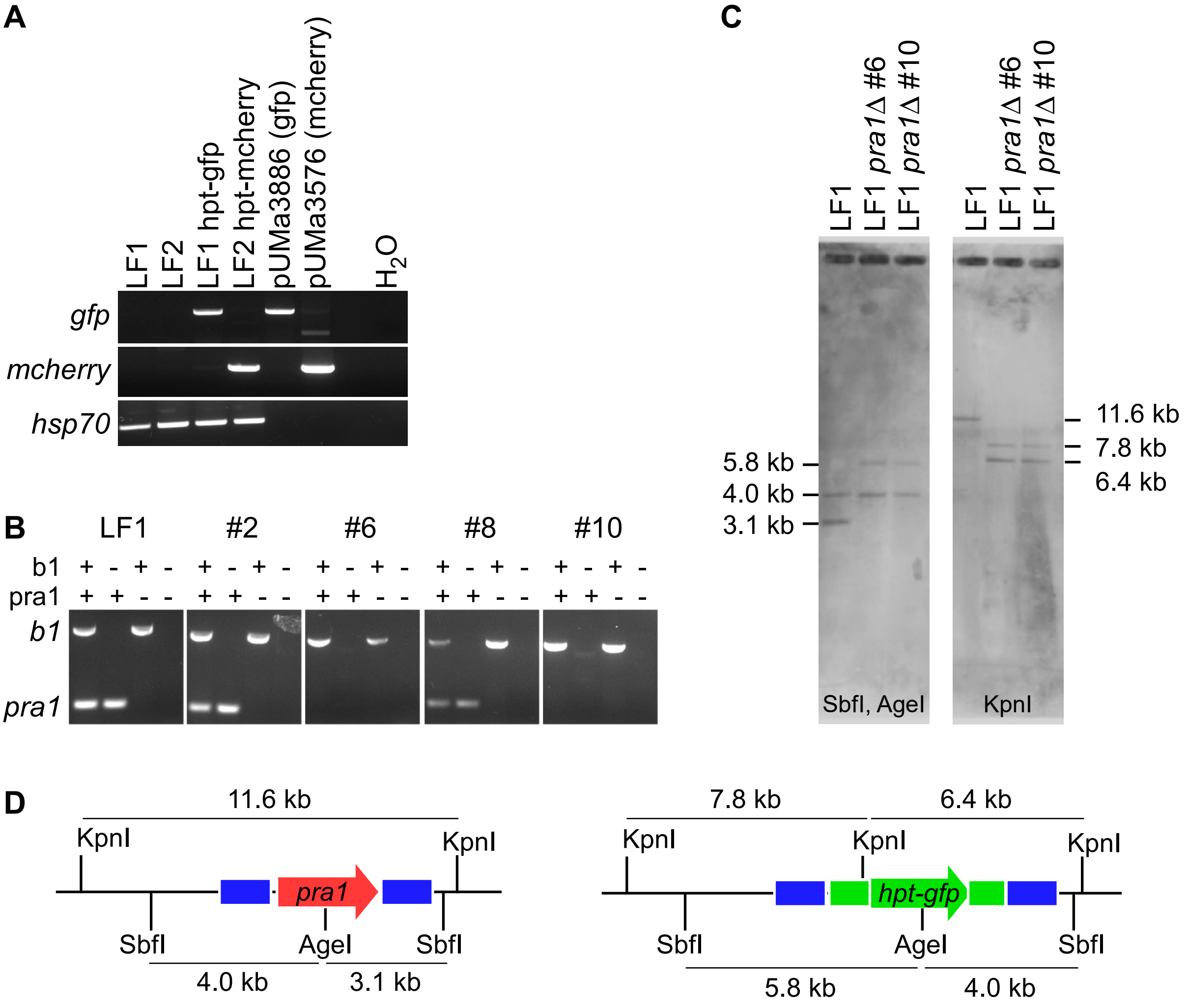

Fig. S5: Confirmation of reporter and deletion strains. (A) Stable integration of the reporter constructs was verified by PCR. Amplification of a 1,1 kb band using gfp-specific primers confirmed the integration only in the hpt-gfp strain, amplification of a 1,1 kb band with mcherry-specific primers confirmed integration only in the hpt-mcherry strain. A 540 bp fragment of the *hps70* gene was amplified as a positive control for gDNA. Wildtype strains and plasmids were used as controls. (B) Deletion of *pra1* was verified in 2 steps. First, absence of the gene was shown in a mulitplex PCR using primers for *pra1* and *b1* as indicated. Both genes are amplified in the wildtype strain LF1 (*pra1*: 150 bp, *b1*: 758 bp), only *b1* is amplified in the deletion mutant candidates #6 and #8, while again both genes are amplifed in strains with ectopic insertion of the resistance cassette as exemplified here in candidate #2 and #8. (C) Second, for two candidates deletion was confirmed by Southern Blot analysis. Genomic DNA was digested with SbfI-HF + AgeI-HF (left blot) or KpnI-HF (right blot). Detection with 1 kb *pra1* flanking regions as probes shows bands of 3.1 kb + 4.0 kb (SbfI + AgeI digest) or 11.6 kb (KpnI) in wildtype strains and 5.8 kb + 4.0 kb (SbfI + AgeI digest) or 7.8 kb + 6.4 kb (KpnI digest) in deletion mutants. (D) Schematic view of the *pra1*-locus and the integration of the deletion cassette into this locus illustrating the restriction sites used for the Southern blot. blue: flanking sequences used for homologous recombination, red: *pra1* coding sequence, green: deletion cassette. Both flanking sequences (blue) were labelled and used as probes for the Southern Blot.
