# Supplementary material for: Genetic Manipulation of the Brassicaceae Smut Fungus Thecaphora thlaspeos
Source: J Fungi (Basel). 2021 Jan 9;7(1):38. doi: 10.3390/jof7010038 (PMC7826943; doi:10.3390/jof7010038)
Supplement: Supplementary file 1 [file jof-07-00038-s001.zip › Supplementary Files_proofs/Protocol S1 plasmid maps.pdf]

Protocol S1

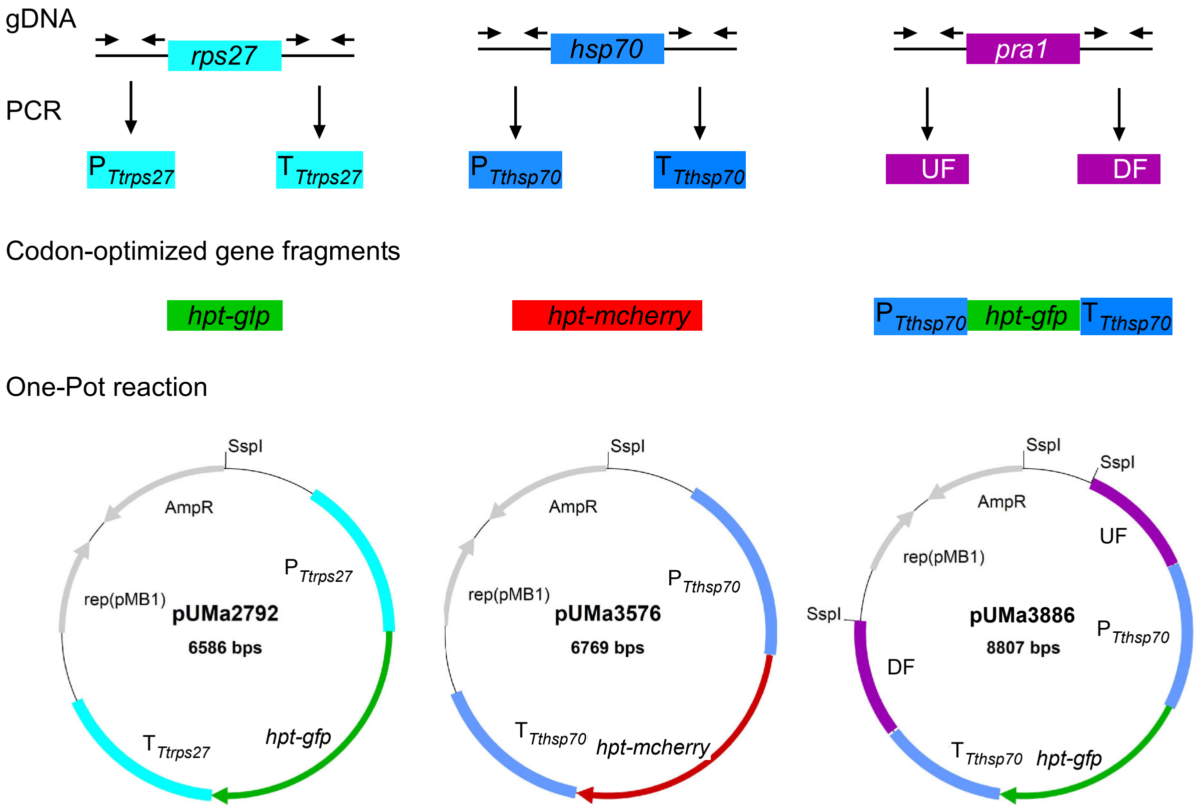

Protocol S1: Golden-Gate assembly of constructs. Exemplarily, the cloning strategy for three constructs is shown. For the reporter constructs, endogenous regulatory sequences were amplified by PCR from genomic DNA extracted from *T. thlaspeos* LF1 1 kb upstream and downstream of the highly expressed genes *hsp70* (THTG_01007) and *rsp27* (THTG_04331). Codon-optimized *hpt-gfp* and *hpt-mcherry* fusion constructs were ordered as gene fragments from IDT, Leuven, Belgium. Plasmids were assembled into a destination vector using Golden-Gate Cloning. For the *pra1* deletion construct, the hpt-gfp-resistance cassette from pUMa2792 was used. 1 kb flanking sequences of *pra1* (THTG_02790) were amplified for homologous recombination. The flanks and the resistance-fusion cassette were assembled by Golden-Gate cloning.
