# Supplementary material for: Genetic Manipulation of the Brassicaceae Smut Fungus Thecaphora thlaspeos
Source: J Fungi (Basel). 2021 Jan 9;7(1):38. doi: 10.3390/jof7010038 (PMC7826943; doi:10.3390/jof7010038)
Supplement: Supplementary file 1 [file jof-07-00038-s001.zip › Supplementary Files_proofs/Protocol S2_Protoplasts and transformation of Thecaphora thlaspeos.pdf]

Protocol S2

Protoplasts of *Thecaphora thlaspeos*

- Inoculate 100 ml of YMPG to an OD_{600} of 0,075 with exponentially growing *T. thlaspeos* culture in a 500 ml baffled flask
- Incubate 18°C, shaking at 200 rpm for 3-4 days until OD_{600} reaches 0,6-0,8
- Harvest cells in a cell strainer (40 µm pore size)
- Wash with 20 ml citrate buffer
- Dissolve 20 mg/ml Glucanex and 10 mg/ml Yatalase in citrate buffer and filter-sterilize dissolved enzyme (9 ml enzyme solution /100 ml culture)
- Transfer the filaments into a 50 ml falcon tube and add 9 ml enzyme solution
- Incubate for 30-60 min at RT → formation of protoplasts is visible already after 10 min
- Add 15 ml of citrate buffer and aliquot in 15 ml falcon tubes, 6 ml each
- Overlay with 5 ml trapping buffer (keep everything on ice from now on)
- Spin 15 min 4°C 4863 g in a swing out rotor
- Collect interphases in a 50 ml falcon
- Mix at least with an equal volume of cold STC buffer
- Spin 10 min 4°C 4863 g in a swing out rotor
- Take off supernatant und re-suspend the pellet in 500µl cold STC buffer
- Prepare 100 µl aliquots in 2 ml reaction tubes
- Proceed with transformation immediately

Transformation of *T. thlaspeos* protoplasts

- Prepare antibiotic bottom-plates (can be prepared the day before):
    - boil up YMPG-REG agar & cool down to 60°C
    - add antibiotic
    - pour 15 ml/plate
    - let it solidify
- Add 15 ml YMPG-REG without antibiotic (prior to transformation)
- Add 1 µl heparin in each transformation tube
- Add linearized DNA (5 µg)
- Incubate 10 min on ice
- Add 500 µl STC/PEG (mix gently)

- Incubate 15 min on ice
- Disperse your transformation on 2 YMPG-REG plates and streak out gently!
- Incubate RT (18°C), wrap with parafilm once they are dry (usually 24 h later)

Media & solutions

**YEPSlight**	800 ml
1.0 % (w/v) Yeast-Extract (Difco)	8.0 g
0.4 % (w/v) Bacto™-Peptone (Difco)	3.2 g
0.4 % (w/v) sucrose	3.2 g

- Dissolve in MilliQ water, aliquot and autoclave for 5 min at 121 ° C

**1x citrate buffer**		500 ml
0.1 M	trisodium citrate 2x H_2O	14.75 g
0.01 M	EDTA (0.5M Stock)	10.00 ml
1.2 M	$MgSO_4$ 7x H_2O	97.88 g

- Dissolve citrate and $MgSO_4$ in MilliQ H_2O
- Then adjust pH to 5,8 with citric acid solution
- Fill up to 490 ml with MilliQ H_2O
- Add EDTA
- Autoclave for 5 min at 121 ° C or filter sterilize

**1x Citric acid solution**		200 ml
0.1 M	citric acid	4.20 g
1.2 M	$MgSO_4$ 7x H_2O	29.58 g

**Trapping buffer**	500 ml
0.6M sorbitol	54.65 g
0.1M Tris/HCl (1M Tris/HCl pH7)	50.00 ml

**YMPG-REG**	800 ml
0.3 % (w/v) Yeast-Extract (Difco)	2.4 g
0.3 % (w/v) Malzextrakt	2.4 g
0.5 % (w/v) Bacto™-Pepton (Difco)	4.0 g
1.0 % (w/v) Glucose	8.0 g
1.0 M sucrose	273.8 g

- In MilliQ water
- Solid medium:
  - + 1.0 % phytagel (4,0 g for 400 ml) or
  - + 0.6 % plant agar (2,4 g for 400 ml
- Aliquot and autoclave for 5 min at 121 °C.
