# Supplementary material for: Genetic Manipulation of the Brassicaceae Smut Fungus Thecaphora thlaspeos
Source: J Fungi (Basel). 2021 Jan 9;7(1):38. doi: 10.3390/jof7010038 (PMC7826943; doi:10.3390/jof7010038)
Supplement: Supplementary file 1 [file jof-07-00038-s001.zip › Supplementary Files_proofs/Tab_S1_protoplasting_pilot.pdf]

Journal of Fungi

MDPI

Table S1. **Pilot study for protoplasting enzyme cocktail.** Various conditions for each enzyme combination were tested mostly in single replicates.

Organism	Enzyme	Buffer composition	Temperature	Incubation time	Yield	Reference
U. maydis	12,5 mg/mL Glucanex	0,02 M citrat-Puffer, 1 M sorbitol	RT	2 h	-	Bösch et al 2016
U. maydis	100 mg/mL Glucanex	0,02 M citrat-Puffer, 1 M sorbitol	RT	2 h	-	Bösch et al 2016
U. maydis/ A. niger	20 mg/mL Glucanex<br>0,015 U/mL Chitinase	0,02 M citrat-Puffer, 1 M sorbitol	RT	up to 20 h	-	Bösch et al 2016,<br>de Bekker et al 2009
U. maydis/ A. niger	20 mg/mL Glucanex<br>0,3 U/mL Chitinase	0,02 M citrat-Puffer, 1 M sorbitol	RT	2 h	+/-	Bösch et al 2016,<br>de Bekker et al 2009
S. indica	20 mg/mL Glucanex	0,02 M MES, 0,05 M CaCl2, 1,33 M sorbitol	RT, 28°C, 37°C	up to 5h	-	Zuccaro et al 2009
S. indica	100 mg/mL Glucanex	0,02 M MES, 0,05 M CaCl2, 1,33 M sorbitol	RT, 28°C, 37°C	up to 5 h	-	Zuccaro et al 2009
U. bromivora	10 mg/mL Glucanex<br>5 mg/mL Yatalase	0,02 M MES, 1 M MgSO4	RT	up to 1,5 h	-	Rabe et al 2016
U. bromivora	**20 mg/mL Glucanex**<br>**10 mg/mL Yatalase**	**0,02 M MES, 1 M MgSO4**	**RT**	**up to 1,5 h**	**+**	**Rabe et al 2016**
