# Supplementary material for: Genetic Manipulation of the Brassicaceae Smut Fungus Thecaphora thlaspeos
Source: J Fungi (Basel). 2021 Jan 9;7(1):38. doi: 10.3390/jof7010038 (PMC7826943; doi:10.3390/jof7010038)
Supplement: Supplementary file 1 [file jof-07-00038-s001.zip › Supplementary Files_proofs/Tab_S2_transformation_constructs_VG.pdf]

Journal of Fungi

MDPI

Table S2 Transformation constructs used for the generation of *T. thlaspeos* strains. * indicates that the plasmid was part of the plasmid mix used in the first transformation.

Vector	Expression cassette						Insertion locus	Selective marker	Enzyme
	**Promoter**	**ID**	**Gene**	**Terminator**	**ID**	**1 kb flanks**			
pUMa2732*	Um Phsp70	UMAG_03791	hpt-gfp	Um Thsp70	UMAG_03791	-	ectopic	hygromycin	SspI HF
pUMa2790*	Tt Phsp70	THTG_01007	hpt-gfp	Tt Thsp70	THTG_01007	-	ectopic	hygromycin	SspI HF
pUMa2792*	Tt Prps27	THTG_04331	hpt-gfp	Tt Trps27	THTG_04331	-	ectopic	hygromycin	SspI HF
pUMa3030*	Tt Phsp70	THTG_01007	hpt-gfp	Tt Trps27	THTG_04331	-	ectopic	hygromycin	SspI HF
pUMa3031*	Tt Prps27	THTG_04331	hpt-gfp	Tt Thsp70	THTG_01007	-	ectopic	hygromycin	SspI HF
pUMa3576	Tt Phsp70	THTG_01007	hpt-mcherry	Tt Thsp70	THTG_01007	-	ectopic	hygromycin	SspI HF
pUMa3886	Tt Phsp70	THTG_01007	hpt-gfp	Tt Thsp70	THTG_01007	*pra1* (THTG 02790)	*pra1*	hygromycin	SspI HF
